# Molecular Structure-Affinity Relationship of Bufadienolides and Human Serum Albumin *In Vitro* and Molecular Docking Analysis

**DOI:** 10.1371/journal.pone.0126669

**Published:** 2015-05-06

**Authors:** Jing Zhou, Guodi Lu, Honglan Wang, Junfeng Zhang, Jinao Duan, Hongyue Ma, Qinan Wu

**Affiliations:** 1 Collaborative Innovation Center of Chinese Medicinal Resources Industrialization, College of Pharmacy, Nanjing University of Chinese Medicine, Nanjing, PR China; 2 College of Basic Medicine, Nanjing University of Chinese Medicine, Nanjing, PR China; 3 Gansu University of Traditional Chinese Medicine, Lanzhou, PR China; University of Quebect at Trois-Rivieres, CANADA

## Abstract

The development of bufadienolides as anti-tumor agents is limited due to poor pharmacokinetic properties regarding drug half-lives and toxicity *in vivo*. These serious factors might be improved by increasing the drug/albumin-binding ratio. This study therefore investigated the relationship between the structural properties of nine bufadienolides and their affinities for human serum albumin (HSA) by a fluorescence spectroscopy-based analysis and molecular docking. Fluorescence quenching data showed that the interaction of each bufadienolide with HSA formed a non-fluorescent complex, while thermodynamic parameters revealed negative *ΔS* and *ΔH* values, corresponding to changes in enthalpy and entropy, respectively. The structural differences between the various bufadienolides markedly influenced their binding affinity for HSA. With the exception of a C = O bond at the C12 position that decreased the binding affinity for HSA, other polar groups tended to increase the affinity, especially a hydroxyl (OH) group at assorted bufadienolide sites. The rank order of binding affinities for drugs with tri-hydroxyl groups was as follows: 11-OH > 5-OH > 16-OH; in addition, 16-acetoxy (OAc), 10-aldehyde and 14-epoxy constituents notably enhanced the binding affinity. Among these groups, 11-OH and 16-acetyl were especially important for a seamless interaction between the bufadienolides and HSA. Furthermore, molecular docking analysis revealed that either an 11-OH or a 16-OAc group spatially close to a five-membered lactone ring significantly facilitated the anchoring of these compounds within site I of the HSA pocket via hydrogen bonding (H-bonding) with Tyr150 or Lys199, respectively. In summary, bufadienolide structure strongly affects binding with HSA, and 11-OH or 16-OAc groups improve the drug association with key amino acid residues. This information is valuable for the prospective development of bufadienolides with improved pharmacological profiles as novel anti-tumor drugs.

## Introduction

Toad venom, called Chansu in China, is a traditional Chinese medicine (TCM) obtained from the skin and parotid venom glands of the toad, including *Bufo bufo gargarizans* Cantor and *B*. *melanostictus* Schneider. In China, toad venom is frequently used as an effective clinical TCM preparation (e.g., as cinobufotalin or Chansu injection) to treat malignant tumors [1]. The therapeutic effect of toad venom stems from its major active ingredients, corresponding to assorted bufadienolides [2] (bufalin, cinobufagin and so on). All major bufadienolides in toad venom exhibit significant anti-tumor activity, including the inhibition of cell proliferation, induction of cell differentiation, induction of apoptosis, disruption of the cell cycle, inhibition of cancer angiogenesis, reversal of multi-drug resistance and enhancement of cytotoxic drug activity [3, 4].

Ideally, anti-tumor drugs should maintain a sufficient concentration in host plasma to kill cancer cells. However, most bufadienolides have short half-lives (the half-life of bufalin is only 0.42 h in rat), and effective drug concentrations cannot be upheld for long periods of time *in vivo* [5]. Furthermore, the bufadienolides cause cardiac toxicities, including arrhythmias and heart dysfunction [6, 7]. Therefore, the short drug half-lives and toxicity are serious factors limiting their development as chemotherapeutic agents in oncology.

Human serum albumin (HSA) is one of the most abundant plasma carrier proteins and plays an important role in the transport and disposition of endogenous and exogenous ligands in the blood [8]. The metabolism of many biologically active compounds (drugs, natural products, etc.) in the body is correlated with their binding affinity toward serum albumin. For example, compounds that are extensively bound to serum albumin will have long plasma half-lives and low clearance (Cl) values [9]. Warfarin, theophylline and verapamil all fall into this category. High plasma protein-binding values may also have an impact on toxicity because the free drug fraction is usually responsible for the toxicological actions [10]. Thus, it is possible to improve the half-lives and safety of bufadienolides by increasing the drug/albumin-binding ratio.

The molecular interactions between HSA and many compounds, such as flavonoids, have been successfully investigated [11, 12]. However, the mode of bufadienolide interaction with HSA has not previously been reported. Therefore, the current study evaluated the structure–affinity relationship of nine bufadienolides isolated from toad venom. The affinity of each drug for HSA was investigated by fluorescence spectroscopy analysis and molecular docking.

## Materials and Methods

### Materials and solutions

HSA (A 8230) was obtained from Sigma Chemical Co. (St. Louis, MO, USA). Its purity was ~96–98%, and its molecular weight was 66,480 Da. ChanSu (the dried secretion of *Bufo bufo gargarizans* Cantor) was utilized as a crude TCM drug for bufadienolide isolation and was purchased from Nanjing Medicinal Material Company (Nanjing, China). A total of nine compounds were isolated from ChanSu by preparative chromatography, and their structures were identified by nuclear magnetic resonance or mass spectrometry as telocinobufagin, resibufogenin, cinobufagin, bufalin, bufotalin, arenobufagin, hellebrigenin, desacetyl-bufotalin and gamabufotalin. The purity of each compound was >95%, as determined by high performance liquid chromatography/ultraviolet light analysis.

Tris-Hcl buffer (0.05 M, pH 7.4) containing 0.1 M NaCl was employed to maintain the pH value and the ionic strength of the HSA solution. The working HSA solution (2.0 × 10^–6^ M) was prepared in the Tris-Hcl buffer and stored in a refrigerator prior to use. A working solution of each bufadienolide (0.1–2.0 × 10^–2^ M) was prepared by dissolving the drug in ethanol. All other reagents and solvents were of analytical grade. All aqueous solutions were prepared by using newly purified ultrapure water.

### Fluorescence measurements

Fluorescence spectra were recorded on a MD SpectraMax 190 fluorescence microplate reader (Molecular Devices, Sunnyvale, CA, USA). The intrinsic fluorescence of the HSA molecule was obtained at 310 nm following excitation at 280 nm, and the emission spectra were read at 300–500 nm. To quantify the potential interaction of the nine bufadienolides with HSA, a 1.0 mL aliquot of 2.0 × 10^–6^ HSA was mixed with 10 μL of each bufadienolide solution (final concentrations, 0–2.0 × 10^–4^ M). All experiments were conducted at three different temperatures (298, 302 and 310 K) with the aid of a water bath set at a constant temperature. By using the decrease in fluorescence intensity as a measure, the association constants (K_a_) were calculated for the bufadienolide/HSA complexes acquired at the three different temperatures. The concentration of each bufadienolide/HSA complex was measured three times, and the experiment was repeated two times.

To illustrate the mechanism of bufadienolide/HSA interaction, fluorescence quenching was determined by applying the Stern-Volmer equation [13–16]:

$${\text{F}}_{0}/\text{F}=1+k_{q}\tau_{0}\left[Q\right]=1+K_{sv}\left[Q\right]$$ (Eq 1)

F_0_ and F are the fluorescence intensities in the absence and presence of the quencher, respectively, *k*_*q*_ is the biomolecular quenching constant, *τ*_*0*_ is the lifetime of the fluorescence in the absence of the quencher, [Q] is the concentration of the quencher, and *K*_*SV*_ is the Stern-Volmer quenching constant, which was measured by linear regression of a plot of F_0_/F against [Q].

The binding constant was calculated according to the double-logarithm equation [14–16], as follows:

$$\log\left[\left({\text{F}}_{0}-\text{F}\right)/\text{F}\right]={\text{logK}}_{\text{a}}+\text{nlog}\left[\text{Q}\right]$$ (Eq 2)

*K*_*a*_ is the binding constant, and n is the number of binding sites per HSA molecule.

To investigate the mode of drug binding to HSA, the temperature-dependence of the binding constant was studied at the three different temperatures described above (298, 302 and 310 K). Thermodynamic parameters were then calculated according to Van’t Hoff plots [17]:

$$\ln\left({\text{K}}_{2}/{\text{K}}_{1}\right)=\left(1/{\text{T}}_{1}-1/{\text{T}}_{2})\;\Delta \text{H}/\text{R}\right)$$ (Eq 3)

ΔG=ΔH−TΔS=−RTlnK (Eq 4)

Constant K corresponds to K_a_ at 298, 302 or 310 K, R is the gas constant, T is the absolute temperature, and ΔH and ΔS refer to the changes in enthalpy and entropy, respectively, during the quenching process.

### Molecular docking

The complex crystal structures of HSA and the HSA ligand, warfarin (Protein Data Bank identification code: 2BXD), were employed to generate the acceptor model for the docking experiment. The three-dimensional structural information was downloaded from the PubChem Public Chemical Database. Molegro Virtual Docker (MVD) software was used for the docking analysis. MVD (http://www.molegro.com/products.php) is a precise, semi-flexible molecular docking program. By using the MolDock (MD) evolution algorithm, MVD predicted the interaction between the ligand and the HSA protein, as well as the intra-molecular interaction energy of the ligand.

The active sites exploited in the docking analysis were defined as a subset region of a 10-Å radius from the centroid of the warfarin ligand. Before screening, the docking protocol was validated by using additional known ligands, such as warfarin and ibuprofen. Every possible docking model was analyzed by checking the MD scores, hydrogen bonds (H-bonds) and known key amino acid residues in the active site [18].

## Results and Discussion

### Quenching effects of bufadienolides on HSA fluorescence spectra

**Fig 1:**
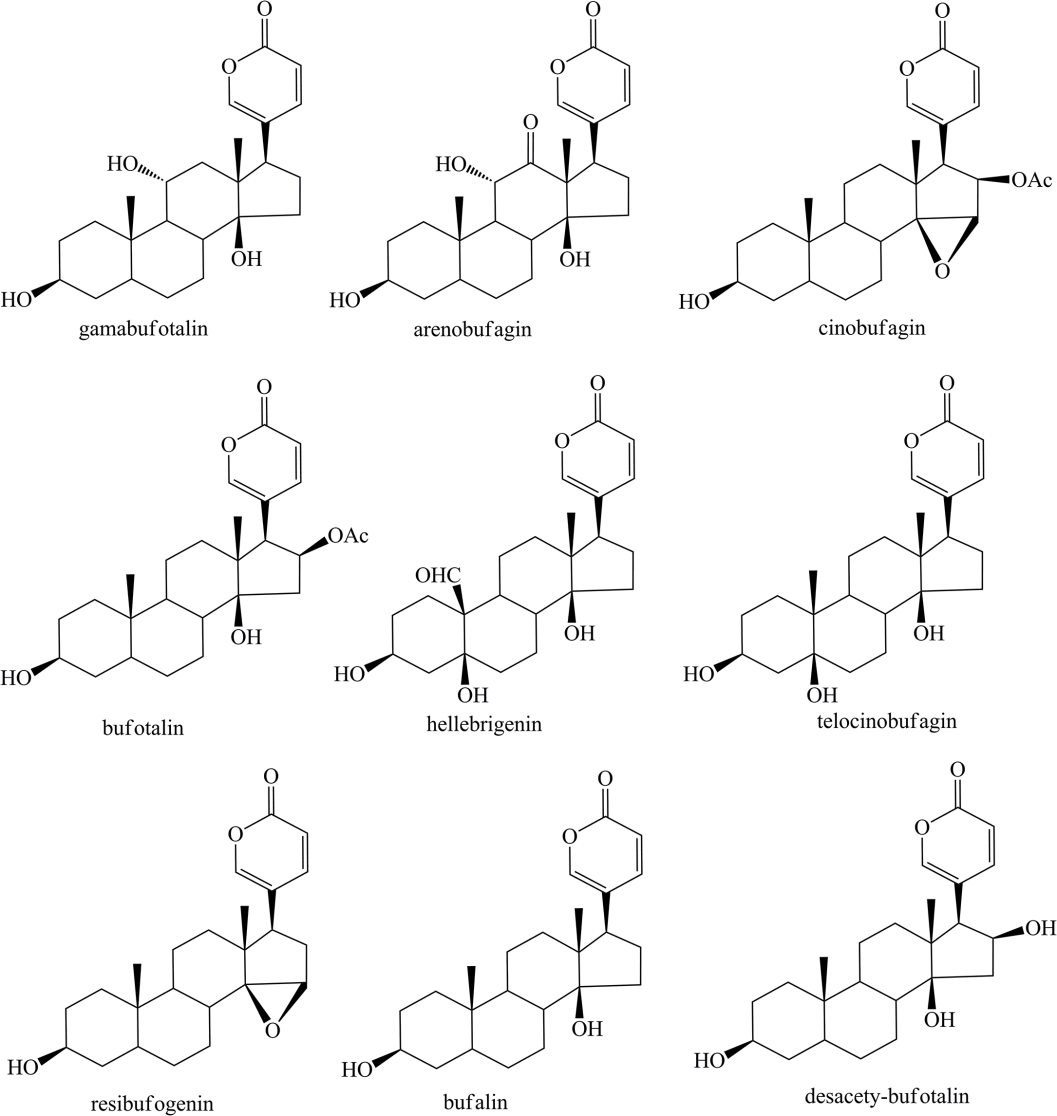
The chemical structure of nine bufadienolides.

**Fig 2:**
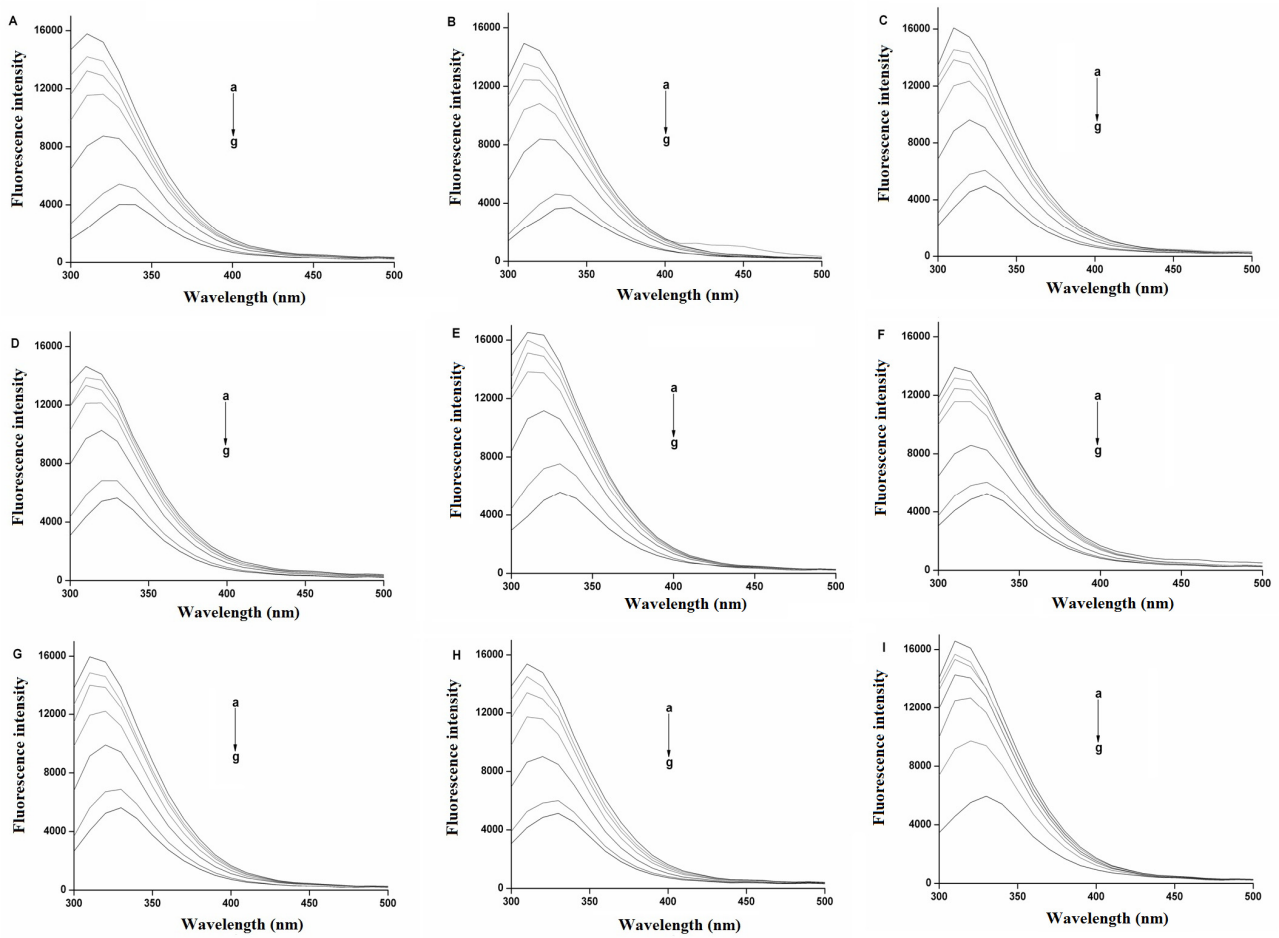
Quenching effect of bufadienolides against human serum albumin (HSA) fluorescence. Experiments were conducted at 298 K. *λex* = 280 nm; HSA, 2.0 × 10^–6^ M; a–g = 0, 10, 20, 40, 80, 160 and 200 × 10^–6^ M for gamabufotalin (A), arenobufagin (B), bufotalin (C), cinobufagin (D), hellebrigenin (E), telocinobufagin (F), resibufogenin (G) and bufalin (H); and a–g = 0, 6.3, 12.5, 25, 50 and 200×10^–6^ M for desacety-bufotalin (I).

The concentration-dependent quenching effects of nine bufadienolides (Fig 1) against the fluorescence intensity of the HSA molecule at 298 K are shown in Fig 2. The fluorescence intensities at 300–500 nm revealed a remarkable decrease with the addition of each of these nine bufadienolides. These results indicate that bufadienolide binding with HSA causes micro-environmental changes in the carrier protein during the generation of the HSA/bufadienolide complex.

HSA fluorescence mainly originates from tryptophan residues, which can be quenched by small molecular probes. Fluorescence quenching corresponds to the decrease in quantum yield of fluorescence from a fluorophore induced by a variety of molecular interactions with the quencher molecules. Fluorescence quenching is accomplished both by collision and complex formation with the quencher [19]. Collision and complex formation forces are dynamic and static, respectively, and can be distinguished by their differing dependence on temperature and viscosity, or preferably by lifetime measurements. Dynamic quenching is due to collisions between quenching agents (drug) and fluorophores (HSA). It is expected to increase the quenching constants with a gradually increasing temperature, because higher temperatures results in larger diffusion coefficients [20]. But the static quenching means that the formation of complex between drug and HSA. And an increase in temperature is likely resulting in a smaller static quenching constant due to the dissociation of weakly bound complexes [21]. To verify that the quenching observed herein represented complex formation between each bufadienolide of interest and HSA, it is important to calculate the value of Ksv, which showed the rate constant of protein dynamic quenching procedure initiated by drugs.

According to Eq 1, we can obtain the biomolecule quenching constants (Table 1) as K_SV gamabufotalin_ = 12.206 × 10^3^ M^-1^, K_SV arenobufagin_ = 8.976 × 10^3^ M^-1^, K_SV cinobufagin_ = 8.149 × 10^3^ M^-1^, K_SV bufotalin_ = 8.118 × 10^3^ M^-1^, K_SV hellebrigenin_ = 7.417 × 10^3^ M^-1^, K_SV telocinobufagin_ = 6.937 × 10^3^ M^-1^, K_SV resibufogenin_ = 6.525 × 10^3^ M^-1^, K_SV bufalin_ = 6.244 × 10^3^ M^-1^ and K_SV desacety-bufotalin_ = 5.474 × 10^3^ M^-1^. The fluorescence lifetime for HSA was set at ~5 ns, based on previous reports [14], and the value of K_Q_ calculated by Eq 1 was as follows: K_Q gamabufotalin_ = 2.441 × 10^12^ M^-1^S^-1^, K_Q arenobufagin_ = 1.795 × 10^12^ M^-1^S^-1^, K_Q cinobufagin_ = 1.630 × 10^12^ M^-1^S^-1^, K_Q bufotalin_ = 1.623 × 10^12^ M^-1^S^-1^, K_Q hellebrigenin_ = 1.483 × 10^12^ M^-1^S^-1^, K_Q telocinobufagin_ = 1.387 × 10^12^ M^-1^S^-1^, K_Q resibufogenin_ = 1.305 × 10^12^ M^-1^S^-1^, K_Q bufalin_ = 1.249 × 10^12^ M^-1^S^-1^ and K_Q desacety-bufotalin_ = 1.095 × 10^12^ M^-1^S^-1^.

**Table 1:** Stern-Volmer quenching constants (*K*_*SV*_) of the interaction of bufadienolides with HSA at different temperatures.

Compounds	T (K)	10^–3^ K_SV_ (M^-1^)	R^2^	SD
	298	12.20	0.9920	0.19
gamabufotalin	302	11.92	0.9939	0.18
	310	11.59	0.9947	0.22
	298	8.97	0.9917	0.18
arenobufagin	302	8.84	0.9932	0.13
	310	8.43	0.9944	0.07
	298	8.14	0.9893	0.21
cinobufagin	302	8.07	0.9898	0.09
	310	7.49	0.9915	0.17
	298	8.11	0.9966	0.26
bufotalin	302	7.78	0.9973	0.28
	310	7.51	0.9878	0.29
	298	7.41	0.9832	0.37
hellebrigenin	302	6.74	0.9917	0.09
	310	6.61	0.9819	0.26
	298	6.93	0.9957	0.16
telocinobufagin	302	6.89	0.9812	0.11
	310	6.84	0.9973	0.21
	298	6.52	0.9860	0.08
resibufogenin	302	6.47	0.9875	0.04
	310	5.84	0.9840	0.04
	298	6.24	0.9818	0.26
bufalin	302	5.71	0.9898	0.29
	310	5.32	0.9924	0.29
	298	5.47	0.9866	0.10
desacety-bufotalin	302	5.46	0.9880	0.03
	310	4.45	0.9983	0.05

R^2^ is the linear correlated coefficient.

From these calculations, we observe that K_Q_ is higher than the limiting diffusion constant, K_dif_ of biomolecules (K_dif_ = 2.0 × 10^10^ M^-1^S^-1^) [22], suggesting the probable quenching mechanism of the binding interaction was static quenching.

To further confirm the quenching mechanism, the linear range of the Stern-Volmer quenching constant was calculated from the slope of the regression curve at 298, 302 and 310 K (Table 1). The quenching constants decreased with increasing temperature, verifying that the discerned fluorescence quenching resulted from a specific interaction between HSA and the bufadienolides.

### The binding constant, *K*_*a*_, and the number of binding sites (n)

**Fig 3:**
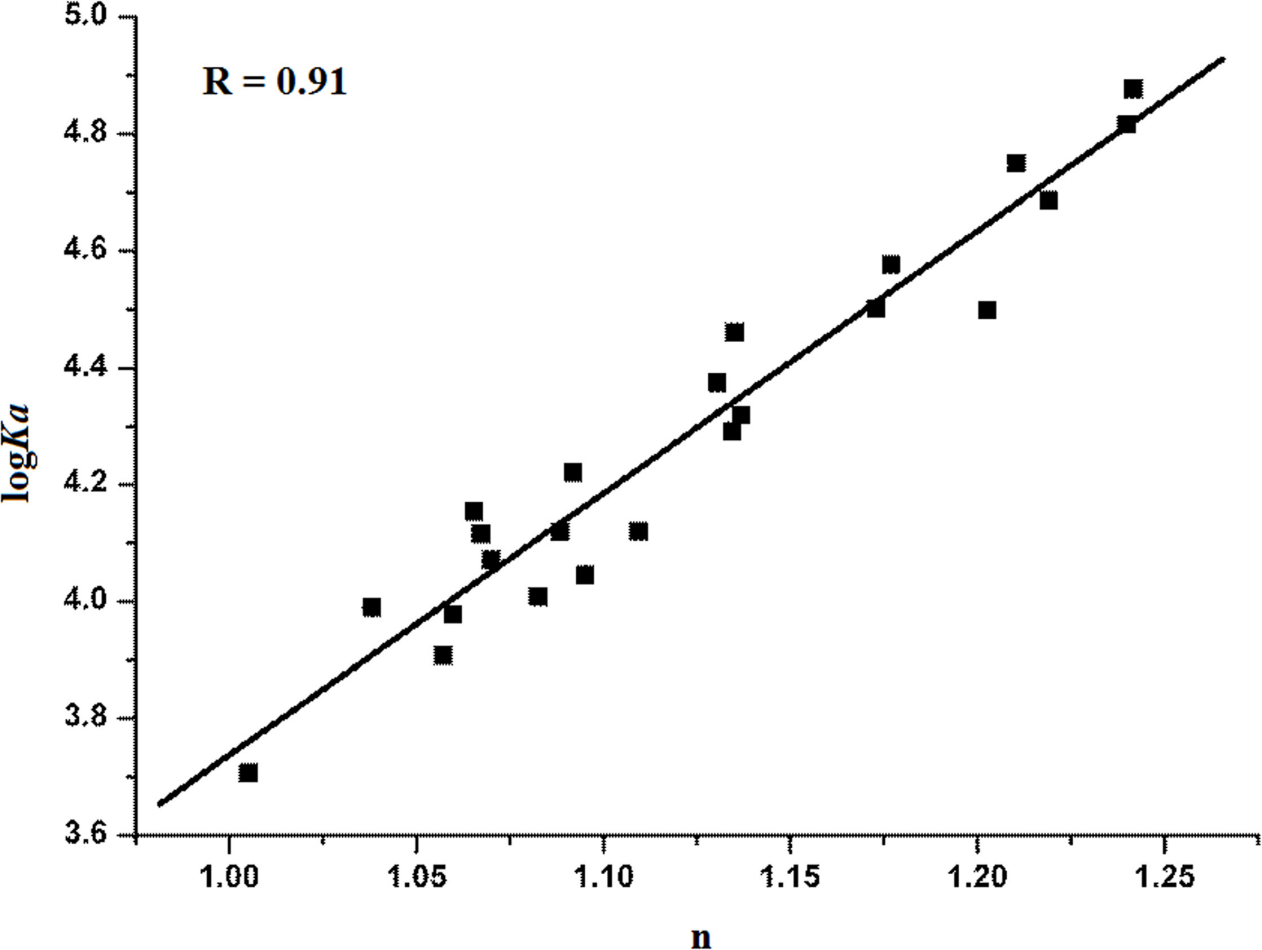
Relationship between bufadienolide binding affinities and the number of binding sites for human serum albumin (HSA). log*Ka* = binding affinity; *Ka* = binding constant; n = number of binding sites; and R = correlation coefficient.

When the possibility of dynamic quenching was excluded, the Ka is further calculated to reflect the binding of drug with HSA. The Ka value could be used to reflect the binding constant of drug-protein complex under the condition of static quenching. Results for the binding constant and the number of binding sites for each drug were calculated according to Eq 2 and are shown in Table 2. The log*K*_*a*_ values are proportional to the number of binding sites (n) (Fig 3), indicating that Eq 1 is suitable as used here for the study of bufadienolide interactions with HSA [23]. K_a_ values for bufadienolide binding to HSA range between 7.5 and 1.0 at pH 7.0 and 298 K, where gamabufotalin and desacety-bufotalin display the highest and the lowest Ka values (× 10^4^ (L·moL ^-1^)), respectively (Table 2). The K_a_ values were determined as follows: gamabufotalin>arenobufagin>cinobufagin>bufotalin>hellebrigenin>telocinobufagin>resibufogenin>bufalin>desacety-bufotalin. These findings imply that bufadienolide quenching effects on HSA fluorescence depend on drug structure.

**Table 2:** The binding constants of bufadienolides on HSA fluorescence.

Compounds	T (K)	n	K_a_ 10^4^ (L·moL ^-1^)	R^2^	SD
	298	1.2	7.53	0.9902	1.62
gamabufotalin	302	1.2	5.61	0.9877	1.72
	310	1.1	2.88	0.9858	2.26
	298	1.2	6.55	0.9975	1.65
arenobufagin	302	1.1	3.77	0.9984	1.75
	310	1.1	2.36	0.9970	1.34
	298	1.2	4.86	0.9967	1.51
Cinobufagin	302	1.1	3.16	0.9954	1.86
	310	1.1	1.88	0.9956	2.44
	298	1.2	3.15	0.9960	1.44
bufotalin	302	1.1	2.03	0.9913	1.18
	310	1.1	1.31	0.9879	2.15
	298	1.1	2.14	0.9929	1.82
hellebrigenin	302	1.1	1.42	0.9960	1.53
	310	1.0	1.11	0.9972	1.10
	298	1.1	2.08	0.9907	1.54
telocinobufagin	302	1.1	1.66	0.9969	2.14
	310	1.1	1.17	0.9924	1.80
	298	1.1	1.95	0.9938	1.13
resibufogenin	302	1.1	1.31	0.9943	1.21
	310	1.1	0.95	0.9956	1.20
	298	1.1	1.66	0.9969	1.43
bufalin	302	1.0	1.30	0.998	1.14
	310	1.0	0.97	0.9977	1.47
	298	1.1	1.01	0.9804	1.23
desacety-bufotalin	302	1.1	0.92	0.9916	1.31
	310	1.0	0.50	0.9920	1.08

R^2^ is the linear correlated coefficient.

The values of n were approximately equal to 1, corresponding to the presence of a single binding site in HSA (most likely Sudlow’s site I within the hydrophobic pocket of subdomain IIA, where Trp213 is located near or actually within the binding site). Our previous study showed that the binding rate of arenobufagin (0.1 μg/ml) to HSA in the presence of 0.05 and 0.1 μg/ml verapamil (a known HSA site I-binding drug [23]) significantly decreased from 61.2 ± 0.2% to 46.7 ± 1.4% and 36.9 ± 3.4%, respectively, as determined by equilibrium dialysis and high performance liquid chromatography/ultraviolet analysis [24]. In the present study, the warfarin (site I probe) was used to perform competitive binding study to determine the binding site of HSA for bufalin using ultrafiltration LC-MS-MS (Text A and Fig A in **S1 Text**). The results showed that warfarin (100 μM) significantly decreased the binding rate of bufalin (30 μM) to HSA (10 μM) by 50%, suggested that bufalin could be removed from the bindings site I by warfarin. So, the bufalin could bind to the site I within HSA. These observations suggest that bufadienolides do in fact bind to site I within HSA.

### Force of the binding interaction between bufadienolides and HSA

Ligands usually integrate with biomolecules via assorted types of binding interactions, including H-bonds, van der Waals forces, hydrophobic bonds and electrostatic interactions. Moreover, certain thermodynamic parameters, notably the enthalpy (*H*) and entropy (*S*) of the binding reaction, are important to confirm the force of drug ligand interactions with biomolecules.

The value of *ΔH* can be obtained from the linear relationship between ln*K* and the reciprocal absolute temperature (*1/T*). The Gibb’s free energy (*ΔG*) and *ΔS* at 298, 302 and 310 K were calculated by using Eqs 3 and 4. From the data in Table 3, we can surmise that the binding process comprises an exothermic reaction accompanied by negative *ΔS* and *ΔH* values. Furthermore, the binding process is spontaneous, as revealed by a negative *ΔG* value. Ross and Subramanian [25] characterized the sign and magnitude of the thermodynamic parameters associated with various kinds of ligand/protein interactions, which may take place during the process of protein association based only on calorimetrically determined enthalpy changes. From this point of view, a negative *ΔS* value generally represents the presence of H-bonds or van der Waals interactions [26]. In addition, we found that *ΔH* term contributed more critically to *ΔG* than the *ΔS* term, so the binding process was enthalpy driven. Accordingly, H-bonds were more likely than van der Waals interactions to play a major role in the bufadienolide/HSA association.

**Table 3:** Thermodynamic Parameters between bufadienolides-HSA interaction at pH 7.4.

Compounds	T(K)	*ΔG* (kJ·mol^-1^)	*ΔH* (kJ·mol^-1^)	*ΔS* (J·mol^-1^·K^-1^)
	298	-27.82 ± 2.63		
gamabufotalin	302	-27.46 ± 2.83	-54.91 ± 9.58	-90.89 ± 0.48
	310	-26.47 ± 3.11		
	298	-27.48 ± 3.37		
arenobufagin	302	-26.46 ± 1.40	-254.56 ± 41.78	-103.33 ± 1.27
	310	-25.96 ±0.76		
	298	-26.74 ± 1.02		
cinobufagin	302	-26.02 ±1.57	-80.32 ± 12.61	-179.81 ± 1.49
	310	-25.38 ±2.30		
	298	-25.66 ± 0.91		
bufotalin	302	-24.91 ±0.42	-81.97 ± 28.01	-188.94 ± 1.96
	310	-24.45 ±1.98		
	298	-24.72 ± 1.48		
hellebrigenin	302	-24.02 ±1.06	-76.85 ± 3.16	-192.13 ± 2.59
	310	-24.01 ±0.25		
	298	-24.64 ± 1.07		
telocinobufagin	302	-24.81 ±1.91	-42.25 ± 17.66	-59.11 ± 0.67
	310	-24.16 ±1.47		
	298	-24.48 ± 0.30		
resibufogenin	302	-23.81 ±0.48	-29.74 ± 17.02	-17.67 ± 1.19
	310	-23.61 ±0.63		
	298	-24.08 ± 0.88		
bufalin	302	-23.79 ±0.34	-45.72 ± 1.79	-72.59 ± 0.88
	310	-23.68 ±1.01		
	298	-22.86 ± 0.51		
desacety-bufotalin	302	-22.59 ±0.67	-42.80 ± 6.27	-66.91 ± 0.10
	310	-22.00 ±2.83		

There are several kinds of groups containing oxygen atoms in bufadienolides to generate the polar interaction with HSA. They include lactone ring, hydroxy (11-OH, 5-OH, 16-OH, 14-OH), 16-acetoxy (16-OAc), 10-aldehyde and 14-epoxy. (1) The six-membered unsaturated lactone ring with 23-O and 21-O played a role in the interaction of bufadienolides with HSA. This group connected with chiral carbon atom C17 is more flexible than other polar groups, and could make specific H-bond interactions with polar residues in the apolar pocket of HSA. (2) The polar substituents in steroid nucleus could generate polar interaction with HSA. 11-OH or 16-OAC paired with spatially neighbor lactone ring substantially increased the binding of bufadienolides via H-bond, but not the 16-OH or 12- (= O) groups. (3) Other polar groups, such as 3-OH, 14-OH (or 14-epoxy), 5-OH and 10-CHO, could produce polar interaction with HSA. In addition, the bufadienolides contain fat-soluble parent nucleus. The hydrophobic interactions via drug aromatic rings as well as the hydrophobic pockets in HSA may be a force of the binding interaction between bufadienolides and HSA.

### Effect of hydroxylation on bufadienolide affinities for HSA

The relationship between drug binding affinities and drug chemical structure can be extracted from Table 2. Considering hydroxyl (OH) groups, the affinities of seven bufadienolides with OH groups for HSA were as follows: gamabufotalin>arenobufagin>cinobufagin>hellebrigenin>telocinobufagin>bufalin>desacety-bufotalin. Along with the two common OH groups at the C-3 and C-14 positions, gamabufotalin and arenobufagin have another OH group at the C-11 position of the C-ring, telocinobufagin and hellebrigenin have another OH group at the C-5 position of the C-ring and desacety-bufotalin has another OH group at the C-16 position of the D-ring. Based on the affinity sequence described above, one may construe that the C-11 OH group assumes a crucial role in increasing drug binding affinity for HSA, while the C-16 OH group negatively affects the binding affinity. Thus, the affinities of bufadienolides with tri-hydroxyl groups for HSA were as follows: OH group at the C-11 position > C-5 position > C-16 position. These findings indicate that different connection locations of OH groups substantially impact bufadienolide binding affinities for HSA.

### Effect of different bufadienolide groups on drug affinities for HSA

When the OH group at the C-16 position in the D-ring is replaced by an acetoxy (OAc) group, the bufadienolide binding affinities for HSA are enhanced. For example, bufotalin and desacety-bufotalin have the same two OH groups at the C-3 and C-14 positions, but bufotalin with 16-OAc in the D-ring has better affinity for HSA than desacety-bufotalin with 16-OH. Furthermore, cinobufagin with 16-OAc exhibits stronger affinity compared with resibufogenin without 16-OAc. These results demonstrate that acetylation of the C16 hydroxyl group increases the drug/HSA association.

We also found that the replacement of the 14-OH group of bufotalin with an epoxy group increased the drug binding affinity for HSA. Cinobufagin with a 14-epoxy group at the C-14,15 position showed stronger interaction with HSA than bufotalin with 14-OH, as did resibufogenin with the 14-epoxy constituent. Therefore, epoxidation of the C14 OH group also enhances the bufadienolide association with HSA.

Hellebrigenin and telocinobufagin have the same three OH groups at the C-3, C-5 and C-14 positions, and the difference between these two compounds is that only hellebrigenin has an aldehyde (CHO) group at C-10. However, the binding affinity of hellebrigenin for HSA is higher than that of telocinobufagin. This suggests that the C-10 CHO group makes a positive contribution to the drug interaction with HSA.

Compared with the functional groups described above, the 12- (= O) group in the C-ring apparently has a negative impact on the binding affinity of bufadienolides for HSA. For instance, arenobufagin with 12- (= O) in the C-ring has a weaker affinity for HSA than gamabufotalin, which lacks this constituent. Hence, it seems that a 12- (= O) group next to the 11-OH group inhibits the drug/HSA association.

### Relationship between bufadienolide chemical structural parameters and affinity for HSA

To further investigate the relationship between drug chemical structure and affinity for HSA, the structural parameters of various bufadienolides (Table 4) were extracted from the PubChem Public Chemical Database (http://pubchem.ncbi.nlm.nih.gov/). Among these structural parameters, the topological polar surface area (TPSA), defined as the sum of the surfaces of all polar atoms in a molecule, showed a positive relationship with bufadienolide log*K*_*a*_ values. From the data in Table 4, it can be seen that bufadienolide affinities increased with increasing TPSA values, indicating that drugs with a greater number of polar groups (i.e., OH groups) are bound more tightly to HSA than drugs with a lower number of polar groups.

**Table 4:** Structural parameters and binding constants of bufadienolides for HSA at 298 K.

Bufadienolides	TPSA	XlogP_3_	H-Bond accepter	H-Bond Donor	lgK_a_
gamabufotalin	87	2	5	3	4.8769
arenobufagin	104	1.7	6	3	4.8164
cinobufagin	85.4	3.3	6	1	4.6867
bufotalin	93.1	2.5	6	2	4.4985
hellebrigenin	104	1.2	6	3	4.3324
telocinobufagin	87	2.5	5	3	4.3195
resibufogenin	59.1	3.7	4	1	4.2904
bufalin	66.8	3.2	4	2	4.2214

**Fig 4:**
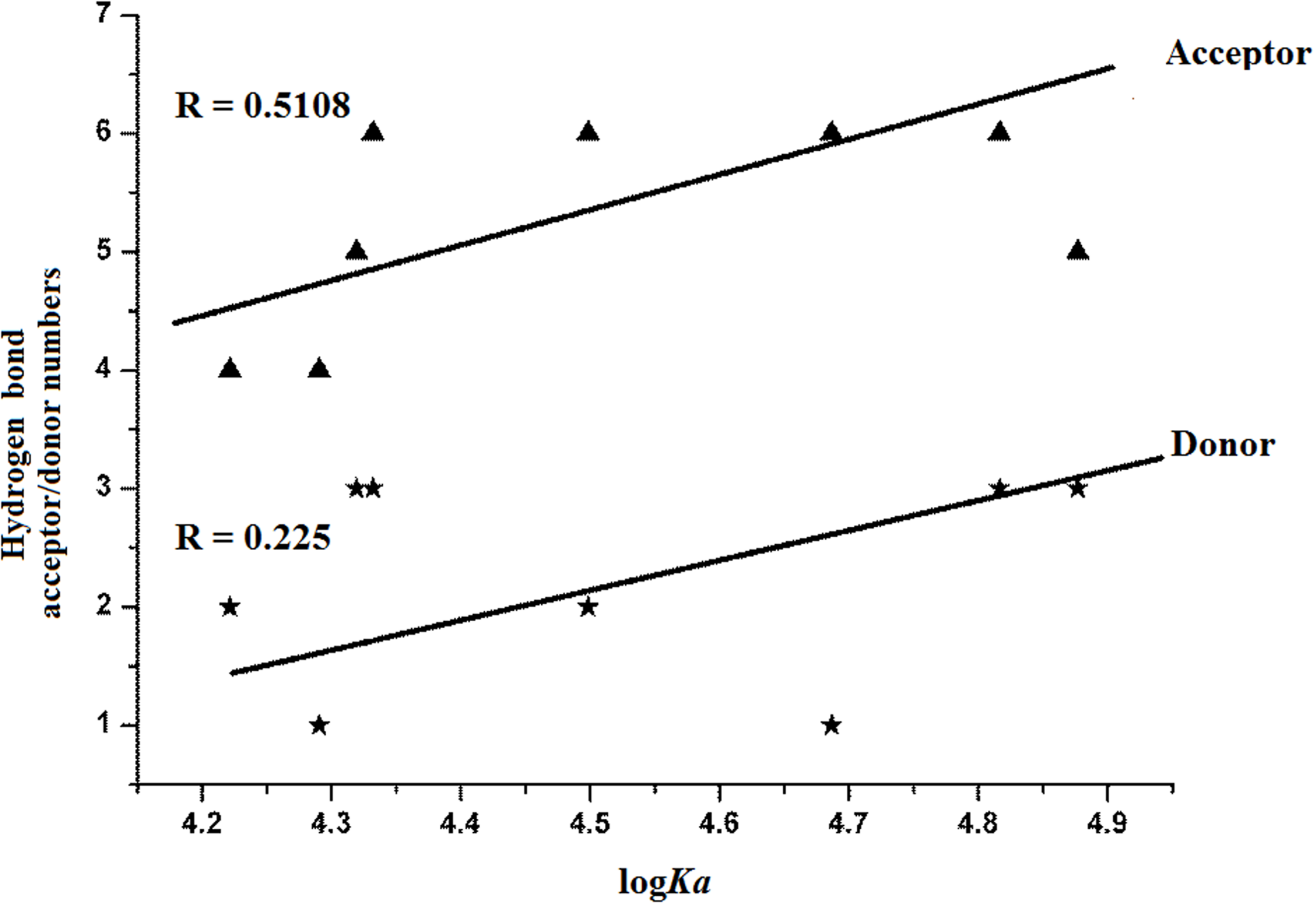
Relationship between hydrogen (H-) bond acceptor/donor numbers and bufadienolide binding affinities for human serum albumin (HSA). The H-bond acceptor/donor numbers were taken from the PubChem Public Chemical Database. log*Ka* = binding affinity; *Ka* = binding constant; and R = correlation coefficient.

Moreover, H-bond acceptor numbers are also positively correlated (correlation coefficient = 0.51) with drug affinity for HSA. The relationship between bufadienolide H-bond acceptor or donor numbers and binding affinity are shown in Fig 4 and clearly indicate that the binding affinities for HSA increased with increasing H-bond acceptor numbers. Therefore, the H-bond force is essential for the bufadienolide/HSA interaction, supporting the above-mentioned hypothesis that H-bonds are a driving factor behind the binding of bufadienolides to HSA.

### Molecular docking of the bufadienolide/HSA complex

**Fig 5:**
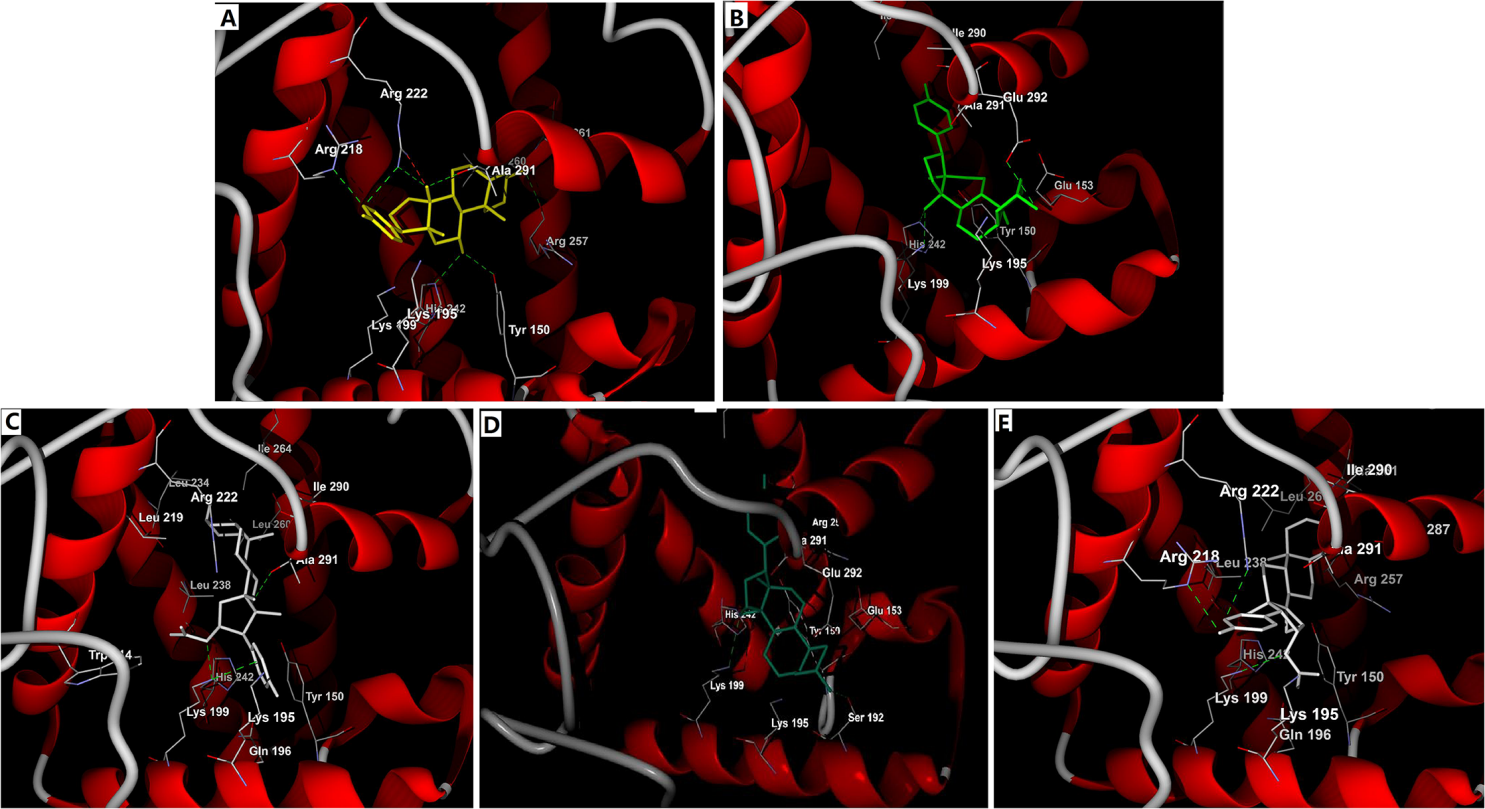
Bufadienolide docking to site I in human serum albumin (HSA). The detailed binding conformations are shown for (A) gamabufotalin, (B) bufalin, (C) bufotalin, (D) resibufogenin and (E) cinobufagin. In each case, the drug is shown as a stick model. Key interaction amino acid residues (with names) are shown as sticks that are color-coded by atom type, and green dashed lines indicate hydrogen bonds (H-bonds).

Molecular docking results are presented in Fig 5 and illustrate that bufadienolides are situated at HSA site I (i.e., the warfarin-binding pocket) within the core of HSA subdomain IIA. Interaction energy scores (MD or re-rank scores) of each bufadienolide/HSA interaction are given in Table 5. These scores show good relevance (-0.85 or -0.81) with the bufadienolide/HSA binding constants (log*K*_*a*_) attained in the fluorescence analysis. Hence, the molecular docking results are useful for analysis of the bufadienolide/HSA interaction mode.

**Table 5:** Molecular docking of various bufadienolides to HSA.

Compounds	MD score (KJ·moL^-1^)	Re-rank score (KJ·moL^-1^)	H-bond score	H-bond receptor of ligand (energy score)	Interaction residues within HSA (energy score)
Gamabufotalin	−113.906	−95.4	−10.8	C14-O (0.15), C11-O (−8.4), C3-O (−2.2), C21-O (−2.4), C23-O (−3.2)	Arg218 (−7.3), Arg222 (−10.5), Arg257 (−13.5), His242 (−7.8), Tyr150 (−9.6), Ala291 (−21.9), Leu238 (−10.5)
Arenobufagin	−111.319	−89.6	−5.0	C14-O (−2.0), C11-O (−4.1), C12-O (6.0), C3-O (−5.2), C21-O (−5.2), C23-O (−6.3)	Lys195 (−15.5), Lys199 (−10.4), His242 (−10.0), Ala291 (−14.0), Leu238 (−6.4), Glu292 (−16.8), Ser192 (−14.3)
Cinobufagin	−121.0	−48.3	−6.5	C14-O (−1.1), C16-O (−3.5), C3-O (−4.4), C21-O (−5.0), C16-O2 (−8.1), C23-O (−6.0)	Arg218 (−9.7), Arg222 (−10.1), Lys195 (−11.3), Lys199 (−12.6), Arg257 (−10.6), His242 (−7.7), Tyr150 (−6.0); Ile290 (−7.6)
Bufotalin	−109.278	−60.5	−5.5	C14-O (4.8), C16-O (−3.7), C3-O (5.1), C21-O (−4.9), C16-O2 (−6.5), C23-O (−5.8)	Arg222 (−4.0), Lys199 (−12.4), His242 (−11.4), Tyr150 (−14.6), Ala291 (−13.8), Leu238 (−15.7)
Hellebrigenin	−95.2	−70.9	−4	C14-O (0.9), C5-O (−2.3), C3-O (−3.0), C6-O (−3.1), C21-O (-4.7), C23-O (-2.7)	Lys199 (−7.6),Arg257 (−12.5), His242 (−7.3), Tyr150 (−7.4), Leu238 (−7.2), Ile290 (−11.8), Ser287 (−9.5)
Telocinobufagin	−91.705	−64.2	−6.7	C14-O (−2.0), C5-O (−2.3), C3-O (−2.7), C21-O (−4.3), C23-O (−5.6)	Arg218 (−7.6), Arg222 (−13.1), Arg257 (−13.5), Ala291 (−14.2), Leu238 (−8.6), Ile290 (−12.2)
Resibufogenin	−102.9	−27.0	−3.7	C14-O (-3.8), C3-O (0.0), C21-O (-3.6), C23-O (−7.5)	Arg257 (−9.1), Tyr150 (−7.9), Ala291 (−9.8), Lys199 (−4.7),Ser192 (−10.7)
Bufalin	−102.3	−24.0	−4.5	C14-O (−6.1), C3-O (−4.2), C21-O (−3.7), C23-O (−7.7)	Ala291 (−11.0), Glu153 (−9.5), His242 (−8.3), Ile290 (−12.1), Lys195 (−10.7), Ser192 (−11.1)
Desacetybufotalin	−73.2	2.167	−3.1	C14-O (−0.5), C16-O (−2.2), C3-O (−4.3), C21-O (−4.4), C23-O (5.7)	Arg222 (−6.3), Tyr150 (−10.8), Ala291 (−14.3), Leu238(−9.5), Ile290 (−7.7)

HSA = human serum albumin; MD score = MolDock score.

Based on the crystallographic analysis of 17 different complexes of HSA with a wide variety of drugs, Ghuman [27] described the precise architecture of HSA site I and identified residues that are key determinants of binding specificity. The interior of site I is predominantly apolar but contains two clusters of polar residues, an inner cluster toward the bottom of the pocket (consisting of Tyr150, His242 and Arg257) and an outer cluster at the pocket entrance (consisting of Lys195, Lys199, Arg218 and Arg222). These key residues make a number of specific H-bond interactions with site I compounds. Furthermore, this pocket is apparently selective for drugs with two electronegative groups (separated by five to six bonds) on both sides of the ligand that can simultaneously interact with the two polar clusters within site I.

Among the nine investigated bufadienolides, gamabufotalin exhibited the best ligand/protein interaction and H-bond energy scores and possesses many important molecular docking features for binding to HSA. Fig 5 shows that gamabufotalin, with two electronegative groups (11-OH/21-O and 3-OH/14-OH), generates six H-bond interactions with critical site I residues and appears particularly well-adapted to the HSA pocket. In the proposed binding model, 11-OH, which has the highest energy score among all gamabufotalin polar groups, played a major role in the H-bond interaction with Tyr150 inner residues. Furthermore, 21-O in the six-membered lactone ring is important for forming H-bonds with outer residues (Arg222 and Arg218). In addition, 3-OH and 14-OH can form H-bonds with Arg257and Arg222, respectively. These findings suggest that the HSA site I pocket is selective for gamabufotalin, with two electronegative groups at a comfortable spatial distance that simultaneously interact with the polar clusters at the pocket entrance and in the bottom of the pocket.

Compared with gamabufotalin, bufalin without the 11-OH group displayed a relatively low energy score for HSA (Table 5). Fig 5 indicates that the molecular binding conformation of bufalin complexed with HSA is completely different from that of gamabufotalin. Due to the absence of the 11-OH group, bufalin cannot form H-bonds between 11-OH or 21-O and polar residues in the site I pocket. Indeed, bufalin only formed two H-bonds via 3-OH/14-OH with His242/Glu292. Moreover, this binding interaction yielded a twisted conformation for bufalin (Fig 5), which is thermodynamically unstable with increased intra-ligand free energy relative to a stretched conformation (i.e., as seen for gamabufotalin). The twisted conformation may further reduce the strength of the bufalin/HSA interaction. This example (bufalin versus gamabufotalin) reveals that 11-OH is important for the binding of bufadienolides to HSA, implying that minor structural modifications could significantly impact the association.

In addition to 11-OH, 16-OAc also participates in the enhanced binding affinity of bufadienolides for HSA. Fig 5 demonstrates that the 16-OAc group of bufotalin forms an H-bond with Lys199, perhaps contributing to the formation of another H-bond via 21-O that is located spatially close to 16-OAc. H-bonds via 16-OAc and 21-O can both stably anchor bufotalin within the HSA site I pocket, imparting higher affinity on bufotalin versus bufalin for the carrier protein. Similar results are observed in Fig 4 for resibufogenin versus cinobufagin. For cinobufagin, the H-bond between 16-OAc and Lys199 is also found in the docking conformation. Like bufotalin, the 21-O group in cinobufagin located in close proximity to the 16-OAc group can form another H-bond with additional polar residues (Arg222), which is helpful for the stable docking of a ligand to a protein. However, there are no spatially adjacent H-bonds in the docking conformation of resibufogenin. Therefore, the examples provided above (bufalin versus bufotalin and resibufogenin versus cinobufagin) illustrate that 16-OAc is also critical for the binding of bufadienolides with HSA.

In addition, the ability of 16-OH or 12- (= O) groups to reduce drug binding affinity for HSA may originate from a steric hindrance effect of 16-OH or 12- (= O), limiting the flexibility of the five-membered lactone ring. Taken together, the docking information provided herein demonstrates that the presence of 11-OH or 16-OAc in close proximity to the lactone ring probably contributes to forming a stable molecular interaction between the bufadienolide of interest and HSA.

## Conclusions

To the best of our knowledge, this is the first report to describe the molecular structure-affinity relationship between bufadienolides and HSA *in vitro* and to thereby offer new insights for the development of bufadienolides as novel anti-tumor drugs. The results of the current fluorescence spectroscopy analysis revealed that bufadienolide compounds induce the fluorescence quenching of HSA due to a specific interaction between the compounds and the protein. The binding force between the drugs and HSA is mainly attributed to H-bond interactions.

Because bufadienolides display unique structural variations, their binding affinities for HSA differ, especially regarding various bufadienolide OH functional groups. The affinities for compounds with tri-hydroxyl groups are as follows: 11-OH > 5-OH > 16-OH. Additional groups connected to the bufadienolide skeleton (i.e., 16-acetyl, 10-aldehyde and 14-epoxy) likewise enhance binding affinity for HSA. According to the molecular docking analysis, the location of either 11-OH or 16-OAc in close proximity to the five-membered acetone ring is probably crucial for H-bonding with Tyr150 and Lys199, respectively, in this way anchoring the bufadienolide within the HSA site I pocket.

In conclusion, the structure of bufadienolides strongly affects their binding with HSA. Drugs with 11-OH or 16-OAc groups may show improved serum albumin binding, contributing to their development as anti-tumor agents.

## Supporting Information

S1 Text Effect of the warfarine on the binding of bufalin to HSA using ultrafiltration LC-MS/MS. (DOCX)
