# Supplementary material for: Molecular Structure-Affinity Relationship of Bufadienolides and Human Serum Albumin In Vitro and Molecular Docking Analysis
Source: PLoS One. 2015 May 6;10(5):e0126669. doi: 10.1371/journal.pone.0126669 (PMC4422520; doi:10.1371/journal.pone.0126669)
Supplement: S1 Text — (DOCX) [file pone.0126669.s001.docx]

**Molecular structure-affinity relationship of bufadienolides and human serum albumin *in vitro* and molecular docking analysis**

Jing Zhou, Guodi Lu, Honglan Wang, Junfeng Zhang, Jinao Duan, Hongyue Ma, Qinan Wu

SUPPORTING INFORMATION (SI)

### SUPPORTING TEXTS

### TEXT A: Binding of bufalin to human serum albumin using ultrafiltration LC-MS-MS

### SUPPORTING FIGURES

Fig. A. Effect of the warfarine on the binding of bufalin to human serum albumin using ultrafiltration LC-MS-MS.

### REFERENCES FOR SUPPORTING INFORMATION

**SUPPORTING TEXTS**

### TEXT A: Binding of bufalin to human serum albumin using ultrafiltration LC-MS-MS

This assay was performed according to the previous reported procedure ^[1,2]^. Bufalin (final concentration: 30 μM) in the presence (or absence) of warfarin (final concentration: 100 μM) was incubated for 2 h at room temperature with 10μM HSA in binding buffer consisting of 50 mM Tris-HCl (pH 7.5) in a total volume of 500 μL. After incubation each mixture was filtered through a 10,000 Da molecular weight cut-off ultrafiltration centrifuge tube (Millipore, Bedford, MA) by centrifugation at 10,000 g for 20min at 4 °C. ultrafiltration was performed three times continuously to remove the unbound compounds. The bound ligands were released by adding 200 μL of methanol/water (90:10; v/v) followed by centrifugation at 14,000 *g* for 20 min. The supernatant was analyzed using LC-MS-MS, which consisted of a Waters ACQUITY UPLC system (Waters, Milford, MA) coupled with a Waters Xevo TQ tandem quadrupole mass spectrometer (Micromass MS Technologies, Manchester, UK). UPLC separation was achieved using an ACQUITY UPLC BEH C18 column (100 mm × 2.1 mm, 1.7μm; Waters) maintained at 40 °C. A gradient elution of solvent A (ultra-pure water contains 0.1% formic acid) and solvent B (acetonitrile) was applied as follows: 0-2min, 20-30%B; 2-6min, 30-35%B; 6-9min, 35-60%B; 9-10min, 60-95%B; 10-11min, 95%B; 11-20min, 95-20%B. The flow rate of the mobile phase was 0.4 mL/min and the injection volume was 2 μl. The column temperature was maintained at 35 °C. Mass spectrometry used an ESI source operated in positive ion mode. The parameters in the source were set as follows: capillary voltage 3.0 kV; campling cone voltage 30V; source temperature 150°C; desolvation temperature 500°C. Data were collected in multiple reaction monitoring (MRM) mode by screening parent and daughter ions.

The results showed that non-specific binding of bufalin to the ultrafiltration apparatus and specific binding to HSA were detected by LC-MS. The analysis of the ultrafiltrates after binding, washing, and release of bufalin from HSA indicated that bufalin bound to HSA but not to blank ultrafiltration membrane. Next, warfarin (site I probe) was used to perform competitive binding study to determine the binding site of HSA for bufalin. The results showed that warfarin (100 μM) significantly reduced the binding rate of bufalin (30 μM) to HSA (10 μM) by 50%, suggested that bufalin could be removed from the bindings site I by warfarin. So, the bufalin could bind to the site I within HSA.

**SUPPORTING FIGURES**


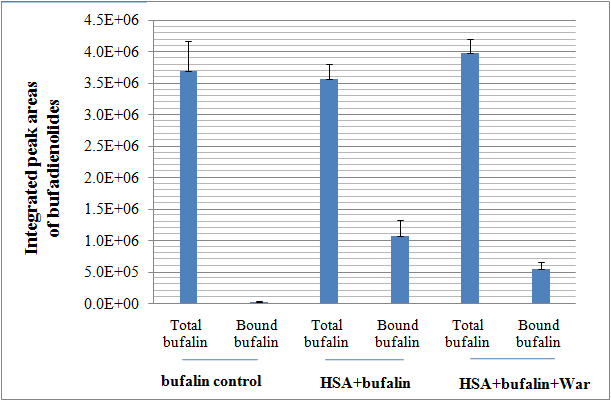


**Fig. A. Effect of the warfarine (War) on the binding of bufalin to human serum albumin using ultrafiltration LC-MS-MS.**

**REFERENCES FOR SUPPORTING INFORMATION**

1. [Choi Y](http://www.ncbi.nlm.nih.gov/pubmed/?term=Choi%20Y%5BAuthor%5D&cauthor=true&cauthor_uid=21192729), [Jermihov K](http://www.ncbi.nlm.nih.gov/pubmed/?term=Jermihov%20K%5BAuthor%5D&cauthor=true&cauthor_uid=21192729), [Nam SJ](http://www.ncbi.nlm.nih.gov/pubmed/?term=Nam%20SJ%5BAuthor%5D&cauthor=true&cauthor_uid=21192729), [Sturdy M](http://www.ncbi.nlm.nih.gov/pubmed/?term=Sturdy%20M%5BAuthor%5D&cauthor=true&cauthor_uid=21192729), [Maloney K](http://www.ncbi.nlm.nih.gov/pubmed/?term=Maloney%20K%5BAuthor%5D&cauthor=true&cauthor_uid=21192729), [Qiu X](http://www.ncbi.nlm.nih.gov/pubmed/?term=Qiu%20X%5BAuthor%5D&cauthor=true&cauthor_uid=21192729), et al. Screening natural products for inhibitors of quinone reductase-2 using ultrafiltration LC-MS. [Anal Chem.](http://www.ncbi.nlm.nih.gov/pubmed/21192729) 2011;83: 1048-1052.

2. Sun Y, Gu C, Liu X, Liang W, Yao P, Bolton JL, et al. Ultrafiltration tandem mass spectrometry of estrogens for characterization of structure and affinity for human estrogen receptors. [J Am Soc Mass Spectrom.](http://www.ncbi.nlm.nih.gov/pubmed/?term=Ultrafiltration+Tandem+Mass+Spectrometry+of+Estrogens+for+Characterization+of+Structure+and+Affinity+for+Human+Estrogen+Receptors) 2005;16: 271-279.
